# Discordance Between Electronic Health Records and Self-Reported Data: Evidence from Traumatic Brain Injury and Colorectal Cancer

**DOI:** 10.3390/healthcare14101337

**Published:** 2026-05-13

**Authors:** Zahra Mojtahedi, Alireza Bolourian, Taylor S. Lane, Monica R. Lininger

**Affiliations:** 1 Center for Community Health and Engaged Research, Northern Arizona University, Flagstaff, AZ 86011, USA; taylor.lane@nau.edu (T.S.L.); monica.lininger@nau.edu (M.R.L.); 2 Department of Computer Engineering, University of Nevada, Las Vegas, NV 89154, USA; bolouria@unlv.nevada.edu; 3 Department of Health Sciences, Northern Arizona University, Flagstaff, AZ 86011, USA; 4 Department of Physical Therapy and Athletic Training, Northern Arizona University, Flagstaff, AZ 86011, USA

**Keywords:** agreement, colorectal cancer, concordance, education, EHR, Medicaid, survey, TBI

## Abstract

**Background/Objectives:** Discordance between electronic health records (EHR) and self-reported survey data may reflect incomplete clinical documentation on the provider side, as well as sociodemographic differences among survey participants. Cancer conditions are frequently reported with the least discordance. Traumatic brain injury (TBI) may be particularly prone to discordance. The aim of this study was to nationally investigate discordance between EHR and self-reported data for TBI and colorectal cancer. **Methods:** This cross-sectional study used data from the national *All of Us* Research Program, including participants with both linked EHR and self-reported survey data. Participants in each condition were stratified into four groups: EHR+/Survey+ (concordant positive), EHR−/Survey− (concordant negative), EHR+/Survey− (discordant), and EHR−/Survey+ (discordant). EHR-documented and survey-reported conditions were compared using a 2 × 2 classification framework to assess concordance. Agreement metrics, including sensitivity, specificity, predictive values, overall concordance/discordance, directional discordance, and Cohen’s kappa, were calculated. Logistic regression models were used to examine the association between the outcomes and sociodemographic factors. Machine learning models additionally investigated the predictive performance of these factors. **Results:** For TBI, concordance between EHR and survey was fair (κ = 0.33), with sensitivity of 60.9% and specificity, 92.9%. In regression models, increasing age was associated with higher odds of both discordant groups (EHR+/Survey− and EHR−/Survey+); lower educational levels and non-White participants had higher odds of discordance specifically in EHR+/Survey− group. Medicaid insurance had higher odds in the EHR−/Survey+ group. In contrast, colorectal cancer showed stronger concordance (κ = 0.66; sensitivity 74.5%; specificity 98.6%) and fewer sociodemographic associations in regression models. The association between race and Medicaid coverage showed a similar pattern to TBI. Machine learning results were also consistent with logistic regression models. **Conclusions:** Concordance between EHR and self-reported data was fair for TBI. Older age, lower education, non-White race, and Medicaid insurance were associated with greater discordance. These sociodemographic patterns were less pronounced in colorectal cancer, except for race and Medicaid insurance. Policies are needed to improve concordance between EHR and self-reported data, particularly across certain sociodemographic groups.

## 1. Introduction

Discordance between electronic health records (EHRs) and self-reported health survey data, defined as when a condition being reported in one source but not the other, represents a persistent challenge, with important implications for epidemiologic data and health services [1,2]. Concordance/discordance between these two data sources are categorized into four groups: EHR+/Survey+ (concordant positive), EHR−/Survey− (concordant negative), EHR+/Survey−, and EHR−/Survey+ (discordant groups reflecting disagreement between EHR documentation and self-report) [2,3,4]. Discordance is associated with various factors, including the completeness of EHR documentation [5] as well as patients’ understanding of their diagnoses for self-reporting [3]. Cancer typically shows high concordance between EHRs and self-reported data, likely because it is consistently documented across multiple encounters and represents a clearly defined diagnosis that is effectively communicated to patients for self-reporting [6]. However, other conditions may be more vulnerable to discordance because of less consistent documentation in the EHR [2,5] or limited patients’ understanding of medical terminology when self-reporting [3]. Traumatic brain injury (TBI) represents one such example [5,7], for which large-scale evidence remains limited. The Centers for Disease Control and Prevention has acknowledged that U.S. TBI estimates are constrained by incomplete data, underscoring ongoing challenges in accurately capturing the true burden of TBI nationwide [8]. The extent of TBI discordance across different data sources and its associated factors may reflect underlying mechanisms of misclassification, such as underdocumentation in clinical records or reporting biases in self-reporting health surveys. Understanding these patterns can help limit systematic bias in epidemiologic estimates and case identification, ultimately improving related health outcomes and policies [2,5].

Prior research suggests that discordance between EHRs and self-reported data is associated with multiple factors, notably individual-level characteristics in addition to the disease itself; however, most of this evidence is based on non-TBI conditions [9,10]. Age influences agreement: older adults may face recall limitations that affect accuracy of self-reported health data [11], while having more healthcare visits increases the likelihood of conditions being captured in EHRs [12]. Gender differences have been observed in some studies [3]. Racial and ethnic differences in concordance have also been reported, with minority populations more likely to experience mismatches between EHR and self-report data, potentially due to differences in healthcare access, quality of care, documentation practices, and patient–provider communication, affecting patients’ understanding of their condition as well as the likelihood of receiving a diagnosis and whether it is recorded in the EHR [9]. Educational level is another reported predictor of agreement [3], likely reflecting differences in health literacy and understanding of diagnostic terminology, with college graduates having significantly higher health literacy compared with other educational levels [13]. Insurance status may also influence concordance, as fragmented and interrupted care is more likely to be associated with incomplete documentation [14]. Additional factors, including disease severity, time since diagnosis, cognitive status, and frequency of healthcare utilization, have also been shown to influence concordance between EHR and self-report data sources [11,12]. More research is needed to clarify the sociodemographic factors associated with TBI to better understand and potentially reduce discrepancies between data sources.

Traumatic brain injury may be particularly vulnerable to discordance across data sources [5,15,16]. The disease, especially in its milder forms, may represent a single historical event that may not be consistently recorded in EHRs [17]. Mild TBIs, including concussions, frequently do not result in hospitalization or formal diagnostic coding, increasing the likelihood of under-documentation within EHR systems [17]. For example, it has been demonstrated 53% of patients meeting the American Congress of Rehabilitation Medicine criteria for TBI did not receive a documented Emergency Department diagnosis of TBI in medical records [5]. Individuals may also report a prior head injury in surveys based on personal interpretation of symptoms, even in the absence of a formal medical diagnosis [15]. Traumatic brain injury may not be clearly communicated or understood from the patient’s perspective, resulting in under or over reporting in survey [15]. A prior study by Bombardier et al. compared self-reported TBI with systematic medical records review by physicians blinded to self-reported TBI among patients with spinal cord injury [15]. They observed moderate sensitivity (83%) and low specificity (51%), and only fair agreement between self-report and medical records, indicating notable discordance between self-reported and documented TBI [15]. Sociodemographic characteristics (e.g., age, race, and education), likely through impacting patients’ understanding of disease reporting, the number of encounters with the healthcare system, and the likelihood of EHR documentation, have also been associated with discordance [2,5]. While more data exists for certain conditions, such as cancer and cardiovascular conditions, in large studies [18,19], limited large-scale data are on TBI are available [15,16,17], highlighting the importance of investigating concordance across data sources and its associated factors for TBI.

Given the reported high discordance in TBI between different data sources [15,16,17], and that existing evidence is largely derived from local or specific clinical settings [15,16,17], further research is needed to better understand these patterns in a large-scale study. Accordingly, the aims of this study were to nationally evaluate concordance between EHR-documented and self-reported TBI, and also to identify sociodemographic factors associated with discordance, and to compare these patterns with colorectal cancer. We hypothesized that TBI would exhibit greater discordance than colorectal cancer. We further hypothesized that older individuals, those identifying as non-White, those with lower educational attainment, and those insured through Medicaid (reflecting disruption in continuity of care [20]) would have higher discordance between EHR documentation and self-reported TBI. Additionally, we expected that the association between sociodemographic factors and discordance would be weaker for colorectal cancer than for TBI.

## 2. Materials and Methods

### 2.1. Study Design, Data Source, and Study Population

This study employed a cross-sectional design using data from the *All of Us* Research Program (AoU) Registered Tier Dataset (Version 8) accessed through the AoU Researcher Workbench. AoU is a nationwide cohort of adults aged 18 years and older, led by the National Institutes of Health (NIH) with the goal of advancing precision medicine research [21]. The program collects EHR, self-reported survey data, saliva samples, genotyping, and other health-related information [21]. It is ongoing with continuous enrollment, in which participants join at different times and complete survey modules throughout their participation rather than during a single, fixed survey period. Their goal is to recruit one million or more participants nationwide, with over 800,000 enrollees so far [22]. AoU uses a uniform, centrally developed survey structure to maintain consistency and comparability of self-reported data across the U.S. [23]. The survey tools are developed through expert review, pilot testing, and alignment across content areas, and they are delivered consistently to all participants using standardized questions and predefined response options [23].

The AoU survey modules used in this study were the Basics (demographics, including age, sex at birth, race/ethnicity, education, and healthcare coverage) and the Personal and Family Health History (TBI history or colorectal cancer history). Cohorts were built within the secure AoU Researcher Workbench through the cohort builder tool by selecting relevant survey questions and combining them with the “Has EHR Data” criterion using a logical AND (Supplementary File, Figure S1), with data linkage performed by the program using unique participant identifiers. Detailed information on cohort construction procedures is available on the AoU Research Program website [22]. Two distinct cohorts, one for TBI and one for colorectal cancer were built by selecting: (1) TBI-related survey questions (concept ID 1740627 or answer concept ID 903095 in response to concept ID 43529272) AND “Has EHR Data”; (2) colorectal cancer-related questions (concept ID 836831 or answer concept ID 903095 in response to concept ID 43528515) AND “Has EHR Data”. The built cohorts included 32,826 participants for TBI and 43,110 participants for colorectal cancer, and all participants identified through the cohort builder were included in the study (Supplementary File, Figure S1). This secondary analysis of de-identified data was determined to be exempt by the Northern Arizona University Institutional Review Board (IRB).

### 2.2. Measures

#### 2.2.1. Outcomes

The outcomes included two concordant (EHR+/Survey+ and EHR−/Survey−) and two discordant (EHR+/Survey− and EHR−/Survey+) categories for each cohort. They were defined based on survey questions and Observational Medical Outcomes Partnership (OMOP) concept IDs in the EHR. The OMOP common data model standardizes disparate healthcare data (e.g., EHR, claims, and registry data) into a unified structure with consistent vocabularies and coding systems, enabling harmonized, reproducible, and large-scale observational analyses across diverse data sources [24].

##### TBI Definition

TBI survey-positive status was defined using the survey question: “Including yourself, who in your family has had traumatic brain injury (TBI)? Select all that apply” (concept ID 1740627). Separate response options include “Self”, “Sibling”, “Father”, “Mother”, “Son”, “Daughter”, and “Grandparent”. Participants who selected “Self” (answer concept ID 1384496) were classified as survey+. Participants who selected only family members (“Sibling”, “Father”, “Mother”, “Son”, “Daughter”, or “Grandparent”; answer concept IDs 1740816, 1740929, 1740803, 1740795, 1740701, and 1740926, respectively) without selecting “self” were categorized as survey−. In addition, individuals who selected “None” (answer concept ID 903095) in response to the question with concept ID 43529272 (any brain and nervous system conditions, self or family members) were also categorized as Survey−. Among these Survey+ and Survey− groups, only participants with available EHR data were included in the study. Participants were categorized as EHR+ for TBI if their EHR contained the parent OMOP concept IDs 4132546 (traumatic brain injury) or 4182419 (late effect of traumatic injury to brain). Participants without any of these concept IDs in their EHR were categorized as EHR−.

##### Colorectal Cancer Definition

Colorectal cancer survey-positive status was defined using the survey question ‘Including yourself, who in your family has had colon cancer or rectal cancer? Select all that apply’ (concept ID 836831), with participants selecting ‘self’ (answer concept ID 43528565) grouped as survey-positive. Participants who did not select “self” for this question but instead indicated other family members (answer concept IDs 43528568, 43528567, 43528569, 43528570, 43528566, and 43528571) were categorized as Survey−. In addition, individuals who selected “None” (answer concept ID 903095) in response to the question with concept ID 43528515 (any cancer, self or family members) were also categorized as Survey−. Among these Survey+ and Survey− groups, only participants with available EHR data were included in the study. Participants were categorized as EHR+ for colorectal cancer if their EHR contained any of the following OMOP concept IDs: 36683531 (malignant neoplasm of colon and/or rectum), 4180790 (malignant tumor of colon), 443381 (malignant tumor of sigmoid colon), 4180792 (malignant tumor of rectosigmoid junction), 435754 (malignant tumor of ascending colon), 443384 (malignant tumor of transverse colon), 443382 (malignant tumor of descending colon), 75512 (carcinoma in situ of colon), and 4180791 (malignant tumor of hepatic flexure). Participants without any of these concept IDs in their EHR were categorized as EHR−.

#### 2.2.2. Predictors

We examined sociodemographic characteristics and health insurance coverage as predictors in the regression models. Participants with missing or unknown responses (prefer not to answer, or I do not know) were retained in the dataset and coded as missing. Predictors included age at consent, sex at birth, race/ethnicity, educational levels, and health insurance types. Age at consent was calculated using date of birth and consent date and modeled as a continuous variable in years. Sex was dichotomized as male or female. Race/ethnicity was categorized as non-Hispanic White and non-White (including Black or African American, Hispanic, Asian, American Indian or Alaska Native, Native Hawaiian/Other Pacific Islander, more than one race, none of these, and other single races). The heterogeneous non-White group was created to avoid unstable estimates due to small sample sizes in subgroups (the sample size in some non-White groups was low, particularly for EHR+/Survey− group [81 participants]). Educational levels were stratified into two groups: ≥college graduate or <college graduate, as previous studies have shown that college graduates have the highest health literacy, with significant differences compared with individuals across all other educational levels [13]. Health insurance was categorized as private insurance (employer-sponsored and directly purchased plans), Medicare/Veterans Affairs (VA), Medicaid, other insurance types, and uninsured. Medicare, VA, and Medicaid are all forms of public health insurance; Medicaid was not combined with Medicare/VA, given its substantially lower continuity of coverage compared with Medicare/VA programs [20,25,26]. Because the capture of health conditions in EHR data is encounter-dependent [12], individuals with Medicare or VA coverage, who typically have more consistent healthcare utilization, are more likely to have conditions documented [25,26] compared with populations experiencing disrupted care, such as Medicaid enrollees [20]. Medicaid coverage (public insurance based on income) is often associated with intermittent enrollment (“churning”) that can disrupt care continuity and reduce consistent healthcare utilization and documentation in EHRs [20].

### 2.3. Statistical Analysis

All analyses were conducted within the AoU Researcher Workbench using Python (version 3.10.16) and Google BigQuery client 2.34.4 on Registered Tier Dataset v8. Continuous variables were summarized as mean ± standard deviation. Categorical variables were calculated as counts and percentages.

#### 2.3.1. Concordance and Discordance Analyses Using a 2 × 2 Classification Framework

EHR-documented and survey-reported TBI were compared using a 2 × 2 classification framework. Participants were categorized as EHR+/Survey+ (concordant positive), EHR+/Survey− (discordant), EHR−/Survey+ (discordant), or EHR−/Survey− (concordant negative). Using EHR documentation as the reference, we calculated sensitivity as true positives (EHR+/Survey+) divided by all EHR+ cases, specificity as true negatives (EHR−/Survey−) divided by all EHR− cases, positive predictive value (PPV) (true positives divided by all Survey+ cases), and negative predictive value (NPV) (true negatives divided by all Survey− cases). We also calculated overall concordance (number of concordant classifications divided by the total sample) and overall discordance (number of discordant classifications divided by the total sample), as well as directional discordance (EHR+/Survey− or EHR−/Survey+ divided by the total sample). Agreement beyond chance was evaluated using Cohen’s kappa (κ), where values of 0–0.20 indicate slight agreement, 0.21–0.40 fair agreement, 0.41–0.60 moderate agreement, 0.61–0.80 substantial agreement, and >0.80 almost perfect agreement [2]. Positive and negative agreement measures were also calculated to assess agreement for concordant positive (EHR+/Survey+) and concordant negative (EHR−/Survey−) classifications. Analyses were separately conducted for TBI and colorectal cancer.

#### 2.3.2. Regression Analyses

Separate logistic regression models (two for TBI and two for colorectal cancer) were conducted to examine directional discordance as outcomes: (Model 1) EHR+/Survey− (coded as 1) vs. EHR+/Survey+ (coded as 0) and (Model 2) EHR−/Survey+ (coded as 1) vs. EHR+/Survey+ (coded as 0). The concordant groups were not combined, as EHR−/Survey− may include both true negatives and missed cases. In secondary data analyses, where external validation is often not feasible, individuals identified as positive in both data sources (EHR+/Survey+) may represent the most accurate classification [17] and would serve as the practical concordant group. Concordant negative classification may still reflect underdocumentation in EHR and low patients’ health literacy [5,27,28,29]. EHR+/Survey− may reflect under-reporting in surveys or inaccurate clinical capture, whereas EHR−/Survey+ may reflect underdocumentation in EHRs or potential overreporting in surveys [5,29]. Therefore, discordant groups were analyzed separately to preserve directional differences and allow clearer interpretation of the asymmetric nature of discordance.

Predictors included age (continuous), sex (female, reference), race (non-Hispanic White, reference), education level (≥college graduate, reference), and insurance category (private insurance, reference). Participants with missing variables were retained in the dataset, coded as missing, and automatically excluded only in logistic regression models via pairwise deletion (complete cases). Given the low rate of missingness across most variables, complete case analysis was considered unlikely to substantially affect the results, although potential bias cannot be excluded. Separate models were run for TBI and colorectal cancer. Adjusted odds ratios (aORs) and 95% confidence intervals (CIs) were reported, with statistical significance determined when the CI did not include 1.

#### 2.3.3. Machine Learning

To supplement the regression analyses, a supervised machine learning model was used to assess performance using the area under the receiver operating characteristic curve (AUC), which measures the model’s ability to distinguish between the groups being compared [30,31]. Specifically, the models examined whether sociodemographic characteristics could distinguish individuals in the discordant groups (EHR+/Survey− or EHR−/Survey+) from those in the concordant positive group (EHR+/Survey+). Logistic regression estimates the direction and magnitude of associations between predictors and outcomes, whereas machine learning methods focus on predictive performance and can capture more complex, including non-linear relationships among variables [30]. We used gradient boosting models (XGBoost), a supervised classification algorithm [30], to evaluate whether sociodemographic characteristics could distinguish participants across the defined groups. Predictors were the same as logistic regression models and included age at consent, sex at birth, race/ethnicity, educational levels, and insurance types.

Model performance was evaluated using AUC, which ranges from 0.5 (no discrimination, meaning the model cannot distinguish between the two groups and performs like random guessing) to 1.0 (perfect discrimination, meaning the model correctly assigns a higher predicted probability to the correct group when two individuals from different outcome groups are compared) [31]. To obtain a stable estimate of predictive performance, we applied 10-fold cross-validation. Feature importance values were calculated to assess the relative contribution of each predictor to model performance, indicating how much each variable contributed to improving model predictions. It is worth mentioning that feature importance values do not provide the direction or magnitude of associations, in contrast to regression models [30].

## 3. Results

### 3.1. Characteristics of Participants

In this study, TBI and colorectal cancer were investigated using the national AoU dataset to evaluate concordance between EHR and self-reported health data. Table 1 summarizes the characteristics of TBI and colorectal cancer participants in AoU who completed the relevant survey questions and had linked EHR data. The cohort for TBI included 32,826 participants: (1) concordant EHR+/Survey+ (784 participants), (2) discordant EHR+/Survey− (504 participants), (3) discordant EHR−/Survey+ (2239 participants), and (4) concordant EHR−/Survey− (29,299 Participants). The cohort for colorectal cancer included 43,110 participants: (1) concordant EHR+/Survey+ (905 participants), (2) discordant EHR+/Survey− (309 participants), (3) discordant EHR−/Survey+ (589 participants), (4) concordant EHR−/Survey− (41,307 participants). Generally, the distribution and characteristics of these four groups regarding sociodemographic characteristics differed, particularly for TBI. Consistent with AoU policy [32], which prohibits the reporting of cell counts < 20 to reduce re-identification risk, the “others”, “uninsured”, or missing categories were combined, when needed, in Table 1.

#### 3.1.1. Sociodemographic Factors in TBI

The distribution of sociodemographic factors in the TBI cohort based on EHR and survey status is presented in Table 1. Participants in the concordant EHR+/Survey+ group were younger (mean age 50.0 years) with a higher percentage of females (61.4%) than the other groups (Table 1). Non-Hispanic White participants were more prevalent among those who self-reported TBI, accounting for 76.5% in EHR+/Survey+ and 76.2% in EHR−/Survey+. In terms of education, ≥ college graduates were most represented in EHR+/Survey+ (55.2%). Insurance patterns also differed: Medicaid insurance was more common in EHR−/Survey+ group, but Medicare/VA was less common in EHR+/Survey− group. Generally, the concordant EHR+/Survey+ TBI group was younger and more likely to be female, White, and college educated, which more closely resembled the discordant EHR−/Survey+ group (except education). However, in terms of health insurance, the concordant EHR+/Survey+ group resembled the EHR+/Survey− group, with a higher proportion of private insurance and Medicare/VA coverage, insurance types which are generally associated with greater continuity of care than Medicaid [20,25,26].

#### 3.1.2. Sociodemographic Factors in Colorectal Cancer

The distribution of sociodemographic factors in the colorectal cancer cohort according to EHR and survey status is presented in Table 1. Differences in sociodemographic distributions were smaller across EHR/Survey concordant and discordant groups for colorectal cancer than for TBI. Educational levels were generally high across groups, with ≥ college graduate education reported by 65.4% of the EHR+/Survey− group. Similar to TBI, non-Hispanic White participants were more common in the EHR+/Survey+ concordance, and Medicaid insurance was also more common in EHR−/Survey+ than in the other groups (Table 1).

### 3.2. Concordance and Diagnostic Performance Between EHR and Survey

#### 3.2.1. TBI Discordance

Concordance between EHR-documented and survey-reported TBI was evaluated using a 2 × 2 classification framework (Table 2). Among 32,826 participants, 1288 (3.9%) had EHR-documented TBI and 3023 (9.2%) self-reported TBI. Using EHR documentation as the reference, the sensitivity of survey-reported TBI was 60.9%, indicating that 784 of 1288 participants with EHR-documented TBI also self-reported TBI, whereas specificity was 92.9%, reflecting that 29,299 of 31,538 participants without EHR-documented TBI also did not report TBI in the survey. The PPV was 25.9%, meaning that only one in four participants who self-reported TBI had corresponding EHR documentation (784 out of 3023), while the NPV was 98.3%, indicating strong agreement for non-TBI classification (29,299 out of 29,803). Cohen’s kappa demonstrated fair agreement beyond chance (κ = 0.33). Overall concordance was observed in 30,083 participants (91.7%), while 2743 (8.4%) demonstrated discordance. Prevalence-adjusted measures further showed a positive agreement of 36.4% and negative agreement of 95.5%, suggesting that concordance was driven primarily by agreement among non-TBI cases. The ratio of EHR-documented to survey-reported TBI prevalence was 0.43 (1288 out of 3023), indicating that EHR documentation captured fewer than half the number of TBI cases identified through self-report.

#### 3.2.2. Colorectal Cancer Discordance

Concordance between EHR-documented and survey-reported colorectal cancer is shown in Table 2. Among 43,110 participants, 1214 (2.8%) had EHR-documented colorectal cancer and 1494 (3.5%) self-reported colon cancer. Using EHR documentation as the reference, the sensitivity of survey-reported colorectal cancer was 74.5%, indicating that 905 of 1214 participants with EHR-documented colorectal cancer self-reported it, whereas specificity was 98.6%, reflecting that 41,307 of 41,896 participants without EHR-documented colorectal cancer did not report it in the survey. The PPV was 60.6%, meaning that approximately three in five participants who self-reported colorectal cancer had corresponding EHR documentation (905 of 1494), while the NPV was 99.3%, indicating strong agreement for absence of colorectal cancer (41,307 of 41,616). Cohen’s kappa demonstrated substantial agreement beyond chance (κ = 0.66). Overall concordance was observed in 42,212 participants (97.9%), while 898 (2.1%) demonstrated discordance. Prevalence-adjusted measures further showed positive agreement of 66.8% and negative agreement of 98.9%, indicating strong agreement for both positive and negative classifications. The ratio of EHR-documented to survey-reported colorectal cancer prevalence was 0.81 (1214 out of 1494), indicating that EHR documentation captured approximately 81% of cases identified through self-report.

### 3.3. Logistic Regression

Table 3 presents the results of logistic regression models examining predictors of the discordant EHR+/Survey− and discordant EHR−/Survey+ groups, each compared with the concordant EHR+/Survey+ group. Separate logistic regression models were conducted for TBI and for colorectal cancer.

#### 3.3.1. TBI

For TBI, increasing age was associated with higher odds of discordance in both the EHR+/Survey− and EHR−/Survey+ groups. Non-White participants and lower educational levels had higher odds of discordance only in EHR+/Survey− (aOR = 2.18, 95% CI = 1.65–2.87 for race; aOR = 1.52, 95% CI = 1.17–1.97 for education) (Table 2). Insurance status also showed an association. Participants with Medicare/VA insurance, compared with those with private insurance, had lower odds of EHR+/Survey− discordance (aOR = 0.41, 95% CI: 0.30–0.58), but Medicaid insurance had higher odds of EHR−/Survey+ discordance (aOR = 1.33, 95% CI: 1.03–1.73).

#### 3.3.2. Colorectal Cancer

For colorectal cancer, several patterns differed from those observed for TBI. Increasing age or having Medicare were not significantly associated with odds of discordance in any of the two models. Lower educational attainment was associated with lower odds of EHR+/Survey− discordance (aOR = 0.62, 95% CI: 0.46–0.84). Similar to TBI, non-White participants had higher odds of being in the EHR+/Survey− discordance (aOR = 1.56, 95% CI = 1.11–2.19), and Medicaid was associated with higher odds of EHR−/Survey+ discordance (aOR = 2.28, 95% CI = 1.51–3.44).

### 3.4. Machine Leaning

To supplement logistic regression models, supervised XGBoost machine learning models were used to evaluate whether sociodemographic characteristics could distinguish individuals in the discordant groups (EHR+/Survey− and EHR−/Survey+) from those in the concordant EHR+/Survey+ group, with performance assessed using the AUC (Table 4). Feature importance values are also displayed in Table 4, representing the relative contribution of each predictor to model performance, with higher values indicating greater influence on model predictions.

#### 3.4.1. Machine Learning: TBI

For TBI, the XGBoost model comparing EHR+/Survey− and EHR+/Survey+ demonstrated moderate discrimination (AUC = 0.71), meaning the model correctly ranked a participant in EHR+/Survey− group higher than a participant in EHR+/Survey+ group about 71% of the time. In this model, race contributed most to model predictions, followed by age, education, insurance type, and sex. However, the model comparing EHR−/Survey+ and EHR+/Survey+ demonstrated weak discrimination (AUC = 0.53). Our machine learning results further supported modeling the discordant groups separately, as TBI showed different discrimination for EHR+/Survey− (AUC = 0.71) and EHR−/Survey+ (AUC = 0.53) compared with the concordant EHR+/Survey+ group, indicating the distinct sociodemographic patterns would have been obscured if discordant groups were collapsed into a binary outcome.

#### 3.4.2. Machine Learning: Colorectal Cancer

For colorectal cancer, the XGBoost model comparing EHR+/Survey− and EHR+/Survey+ showed weak discrimination with AUC = 0.59, indicating the model correctly ranked a participant in EHR+/Survey− group higher than a participant in EHR+/Survey+ group about 59% of the time. Age contributed most to the model, followed by education, insurance type, race, and sex. The model comparing EHR−/Survey+ and EHR+/Survey+ showed weak discrimination (AUC = 0.54), with age and insurance type contributing most to model predictions, followed by race, education, and sex.

## 4. Discussion

Discordance between EHR and self-reported health data has been reported in prior studies, particularly for conditions that are variably documented or depend on patient interpretation [5]. In our study, TBI demonstrated relatively low concordance between EHR and self-report data across all metrics. In contrast, colorectal cancer showed substantially higher concordance, suggesting more complete documentation and clearer patient understanding of diagnosis. Logistic regression and machine learning analyses also demonstrated that discordance was associated with sociodemographic characteristics, with varying magnitudes across factors and groups.

Our findings on concordance between EHR and self-reported data are consistent with prior studies examining TBI/injury-related conditions and cancer; for example, recently, Bombardier et al. reported a sensitivity of 83%, specificity of 51%, PPV of 46%, and κ = 0.29, demonstrating fair agreement and substantial higher TBI cases by self-reporting compared with EHR [15]. Here, for TBI, sensitivity was 60.9%, and specificity was 92.9%, with a notably low PPV of 25.9% despite a high NPV of 98.3%. Cohen’s kappa indicated only fair agreement (κ = 0.33). The EHR-to-survey positivity ratio was 0.43, meaning that the EHR captured fewer than half of the cases identified by self-reporting, suggesting possible underdocumenting in the EHR relative to survey data. This highlights a conceptual limitation in using EHR as reference (like our study), particularly for conditions such as TBI with possible underdocumentation [5]. While EHR data provides clinical documentation, they may not fully capture all prior or less severe events. Therefore, measures such as sensitivity and PPV should be interpreted cautiously, as they may reflect limitations of the reference source. In contrast to TBI, colorectal cancer has demonstrated substantially higher concordance both in prior studies [18] and in our findings. A recent colorectal cancer study reported sensitivity of 78.6%, specificity of 99.7%, PPV of 52.4%, NPV of 99.9%, and κ = 0.63 [18]. Here, sensitivity reached 74.5%, specificity 98.6%, PPV 60.6%, and NPV 99.3%, and concordance was substantial (κ = 0.64). Together, these results reinforce that TBI is considerably more prone to discordance across data sources compared with cancer. While these findings are consistent with differences in disease characteristics and reporting, alternative explanations should also be considered. Differences in clinical coding practices and care pathways may contribute to the observed patterns. For example, colorectal cancer is typically managed within structured diagnostic and treatment pathways, leading to more consistent coding and documentation, whereas TBI may be variably documented across care settings, particularly in mild cases or when care is not sought. Variability in care pathways, including emergency versus outpatient management and limited follow-up, may further contribute to inconsistent documentation.

In our study, the distribution of sociodemographic factors differed based on EHR and survey groups, particularly for TBI. In TBI, EHR+/Survey+ group, likely the most accurate one [29], was younger, more educated, more likely to be non-Hispanic White, and less likely to have Medicaid health insurance. Increasing age was associated with higher odds of discordance (both EHR+/Survey− and EHR−/Survey+). Lower educational levels and being non-White were associated with higher odds of discordance only in EHR+/Survey− group; Medicare/VA and Medicaid insurance (vs. private insurance) were associated with lower and higher odds of discordance in EHR+/Survey− and EHR−/Survey+, respectively. Similar associations of age and educational levels were previously reported in certain conditions, such as cardiometabolic diseases [33]. Age may influence discordance through differences in recall and healthcare exposure. Older individuals might face recall of past conditions in surveys but may have had more opportunities for diagnoses to be recorded in the EHR [33]. Lower education may be associated with higher discordance due to differences in health literacy [33], which can affect understanding of diagnoses in surveys and may help explain our findings of its association with EHR-only positivity. Education can also affect engagement with healthcare systems where conditions are documented. Individuals with lower educational levels may also have less consistent access to care, increasing the likelihood that conditions are not recorded in the EHR [33]. Race may impact provider–patient communication and patients’ understanding of their diagnoses [9], which may partly explain our findings of its association with the EHR+/Survey− group. Whether this association was influenced by language barriers was not investigated in the present study. Insurance status can affect discordance in multiple ways. Individuals with Medicare and VA might have less fragmented and more consistent interactions with healthcare systems for chronic care, which may increase the likelihood of EHR documentation [25,26]. In contrast, Medicaid coverage is often intermittent, which can lead to reduced continuity of care and fewer interactions with the healthcare system, potentially resulting in underdocumentation of diseases [20], despite being reported in survey. In our study, fewer sociodemographic factors were associated with discordance in colorectal cancer compared to TBI, likely reflecting more standardized clinical documentation and greater patients’ understanding of the disease. Similarly, prior studies have reported limited sociodemographic variation based on discordance when cancer was examined alongside other conditions [30]. Another study reported that kappa values (agreement scores) for colorectal cancer were higher among individuals with lower educational levels [29], consistent with our findings. The underlying reasons remain unclear; however, colorectal cancer has been noted to have a lower concordance rate compared with other cancer types [29]. The association between race and Medicaid coverage in colorectal cancer followed a similar pattern to that observed for TBI, indicting their association might be independent of the disease itself. Machine learning models further supported the regression findings, showing that the sociodemographic factors examined here had lower predictive ability (lower AUC) for colorectal cancer than for TBI, particularly in the EHR+/Survey− comparison. For both TBI and colorectal cancer, the EHR−/Survey+ models had lower AUC than the EHR+/Survey− models, indicating that EHR−/Survey+ discordance may be less associated with sociodemographic factors and more with non-sociodemographic factors or those related to EHR documentation, such as healthcare utilization (e.g., number of visits), which were not examined in this study.

The findings of this study might have important implications for clinical practice and health policies. The substantial discordance between EHR-documented and self-reported TBI suggests that relying on a single data source may lead to misclassification, particularly among specific sociodemographic groups. Policies that depend primarily on EHR data for surveillance, resource allocation, or risk stratification may underestimate the true burden of TBI, especially among older individuals and Medicaid enrollees. Medicaid enrollees, irrespective of disease (TBI or colorectal cancer), had higher odds of survey-only positive classification, suggesting lower EHR documentation and underscoring the need for policies to improve documentation in this population. Incorporating self-reported data into clinical screening and public health monitoring frameworks could improve case identification and better capture lifetime TBI history, which may often not be consistently documented in EHR [5]. Additionally, efforts to standardize TBI documentation in EHR systems and enhance provider recognition of the importance of recording remote injury history may reduce discordance. Given that TBI is associated with long-term health consequences [34], improving its documentation is critical for informing prevention strategies, and accurately identifying at-risk populations for targeted interventions.

This study has several limitations. Not all participants in the AoU cohort have linked EHR data, which may introduce selection bias. Moreover, differences in how conditions are defined and reported across data sources may contribute to discordance; for example, TBI is reported as a lifetime history in surveys but may not be consistently coded in longitudinal clinical records, whereas colorectal cancer is more likely to be formally diagnosed and documented. We also did not examine TBI severity subtypes (e.g., mild vs. moderate/severe TBI) nor subtypes of colorectal cancer, such as in situ diseases. Differences in severity/subtypes may influence both documentation in EHR and self-reporting behavior. For example, individuals may interpret TBI as a concussion or perceive in situ colorectal cancer as benign, which could contribute to misclassification and affect concordance between data sources. Although we adjusted for key sociodemographic factors, residual confounding may remain due to unmeasured variables such as healthcare utilization, access to care, and disease severity, which may influence both reporting and documentation. These variables were not included in our models primarily due to limitations in their availability and consistency within the AoU dataset. For example, healthcare utilization is not uniformly captured across participants, and disease severity and timing of diagnosis are not consistently available or standardized across both EHR and survey data sources. Our study is also limited due to its reliance on structured EHR data. A substantial portion of clinically relevant information is often recorded in unstructured free-text fields [35], which were not available for analysis in this study. This is particularly relevant for conditions such as TBI, where documentation may be incomplete or inconsistently coded but still present in narrative clinical notes. As a result, the use of structured data alone may underestimate clinical documentation [35] and contribute to the observed discordance between EHR and self-reported data. Another limitation relates to the use of complete case analysis, which may introduce selection bias if data are not missing at random, potentially affecting the magnitude and direction of associations, although missingness was low in our sample. Using EHR as the reference source is also a limitation, particularly for potentially underdocumented conditions such as TBI [5]. Accordingly, sensitivity and PPV should be interpreted cautiously. Finally, the AoU cohort, although it is national, is based on voluntary participation and rolling enrollment, which may limit generalizability.

## 5. Conclusions

In conclusion, here, discordance between EHR documentation and self-reported survey data varied by condition, with substantially lower concordance observed for TBI compared with colorectal cancer. TBI showed fair concordance, reflecting its possible susceptibility to under-documentation in EHR and variability in self-report, whereas colorectal cancer demonstrated substantially higher concordance, consistent with more standardized diagnostic and recording practices and greater patients’ understanding of the disease. Discordance in TBI was associated with sociodemographic factors, whereas colorectal cancer was less associated with the same factors. Incorporating both structured and unstructured EHR data, including the use of natural language processing approaches, may provide a more comprehensive representation of patient conditions and reduce apparent discordance between data sources. Future studies should explore subtype-specific concordance and incorporate measures of healthcare utilization, disease severity, and temporal information to better identify the underlying mechanisms of discordance between EHR and self-reported data.

## Figures and Tables

**Table 1:** Characteristics of participants across study groups according to survey and electronic health (EHR) status (*All of US* research program).

	Traumatic Brain Injury (N = 32,826)				Colorectal Cancer (N = 43,110)			
Characteristic	EHR+/Survey+ (N = 784)	EHR+/Survey− (N = 504)	EHR−/Survey+ (N = 2239)	EHR−/Survey− (N = 29,299)	EHR+/Survey+ (N = 905)	EHR+/Survey− (N = 309)	EHR−/Survey+ (N = 589)	EHR−/Survey− (N = 41,307)
Age, mean (SD)	50.0 (14.7)	53.5 (17.7)	51.5 (14.8)	53.5 (16.8)	63.1 (11.8)	62.9 (11.5)	63.2 (12.1)	53.3 (16.3)
Age, missing	0	0	0	0	0	0	0	0
Sex at birth								
Female	481 (61.4)	294 (58.3)	1313 (58.6)	16,528 (56.4)	490 (54.1)	177 (57.3)	323 (54.8)	26,757 (64.8)
Male	299 (38.1)	205 (40.7)	907 (40.5)	12,612 (43.0)	408 (45.1)	130 (42.1)	260 (44.1)	14,294 (34.6)
Missing	4 (0.5)	5 (1.0)	19 (0.8)	159 (0.5)	7 (0.8)	2 (0.6)	6 (1.0)	256 (0.6)
Race/Ethnicity								
Non-Hispanic White	600 (76.5)	301 (59.7)	1706 (76.2)	16,522 (56.4)	698 (77.1)	221 (71.5)	427 (72.5)	27,362 (66.2)
Non-White	174 (22.2)	197 (39.1)	509 (22.7)	12,330 (42.1)	189 (20.9)	81 (26.2)	147 (25.0)	13,424 (32.5)
Missing	10 (1.3)	6 (1.2)	24 (1.1)	447 (1.5)	18 (2.0)	7 (2.3)	15 (2.5)	521 (1.3)
Education								
(≥college graduate	433 (55.2)	268 (53.2)	1110 (49.6)	15,021 (51.3)	497 (54.9)	202 (65.4)	324 (55.0)	22,986 (55.6)
<college graduate	349 (44.5)	223 (44.2)	1101 (49.2)	13,750 (46.9)	392 (43.3)	105 (34.0)	251 (42.6)	17,719 (42.9)
Missing	2 (0.3)	13 (2.6)	28 (1.3)	528 (1.8)	16 (1.8)	2 (0.6)	14 (2.4)	602 (1.5)
Insurance								
Private	254 (32.3)	186 (36.9)	654 (29.2)	12,266 (41.9)	285 (31.5)	108 (35.0)	145 (24.6)	17,338 (42.0)
Medicare/Veteran Affairs	293 (37.4)	171 (33.9)	857 (38.3)	9017 (30.8)	437 (48.3)	143 (46.3)	293 (49.7)	12,729 (30.8)
Medicaid	122 (15.6)	82 (16.3)	427 (19.1)	4330 (14.8)	65 (7.2)	26 (8.4)	80 (13.6)	5416 (13.1)
Others	39 (5.0) *	32 (6.4) *	52 (2.3)	828 (2.8)	26 (2.9) *	32 (10.3) **	27 (4.6) *	1085 (2.6)
Uninsured	− ***	−	72 (3.2)	1461 (5.0)	−	−	−	1546 (3.7)
Missing	76 (9.7)	33 (6.5)	177 (7.9)	1397 (4.8)	92 (10.2)	−	44 (7.5)	3193 (7.7)

* The “Others” and “Uninsured” categories were merged to ensure cell sizes ≥ 20, consistent with *All of Us* policy to minimize the risk of identification [32]; ** Counts from the “Missing” category were added to “Others” and “Uninsured” to ensure cell sizes ≥ 20. *** Counts from this cell were combined to “Others” cell.

**Table 2:** Concordance between EHR-documented and self-reported data in traumatic brain injury (TBI) (N = 32,826) * and in colorectal cancer (N = 43,110) **.

	TBI			Colorectal Cancer		
Data Source	EHR+	EHR−	Total	EHR+	EHR−	Total
**Survey+**	784	2239	3023	905	589	1494
**Survey−**	504	29,299	29,803	309	41,307	41,616
**Total**	1288	31,538	32,826	1214	41,896	43,110

* Diagnostic performance measures: Sensitivity, 60.9%; Specificity, 92.9%; Positive Predictive Value, 25.9%; Negative Predictive Value, 98.3%; Cohen κ, 0.33; Positive Agreement, 36.4%; Negative Agreement, 95.5%; EHR-to-Survey Positivity Ratio, 0.43. ** Diagnostic performance measures: Sensitivity, 74.5%; Specificity, 98.6%; Positive Predictive Value, 60.6%; Negative Predictive Value, 99.3%; Cohen κ, 0.66; Positive Agreement, 66.8%; Negative Agreement, 98.9%; EHR-to-Survey Positivity Ratio, 0.81.

**Table 3:** Adjusted odds ratios (aOR) and 95% confidence intervals (95% CI) for sociodemographic factors associated with directional discordance (EHR+/Survey− or EHR−/Survey+) compared with EHR+/Survey+ concordance.

	TBI aOR (95% CI)		Colorectal Cancer aOR (95% CI)	
Factors	EHR+/Survey−	EHR−/Survey+	EHR+/Survey−	EHR−/Survey+
Age (per year)	1.03 (1.02–1.04)	1.01 (1.00–1.02)	1.00 (0.99–1.02)	1.00 (0.99–1.01)
Sex (Ref, female)				
Male	0.99 (0.77–1.28)	1.02 (0.85–1.22)	0.84 (0.63–1.11)	0.89 (0.71–1.12)
Race/Ethnicity (ref, Non-Hispanic White)				
Non-White	2.18 (1.65–2.87)	0.93 (0.76–1.15)	1.56 (1.11–2.19)	1.10 (0.83–1.46)
Education (Ref, ≥college graduate)				
<college graduate	1.52 (1.17–1.97)	1.18 (0.99–1.41)	0.62 (0.46–0.84)	0.90 (0.71–1.15)
Health insurance (Ref, private)				
Medicare/VA	0.41 (0.30–0.58)	0.93 (0.74–1.16)	0.89 (0.61–1.32)	1.29 (0.95–1.76)
Medicaid	0.74 (0.51–1.07)	1.33 (1.03–1.73)	1.16 (0.68–1.99)	2.28 (1.51–3.44)
Uninsured	0.72 (0.34–1.52)	1.48 (0.86–2.56)	1.50 (0.53–4.3)	1.65 (0.66–4.12)
Other	0.70 (0.33–1.45)	0.91 (0.53–1.56)	0.20 (0.03–1.54)	2.25 (1.06–4.77)

TBI, traumatic brain injury; EHR+, Electronic health records contained the documentation of the condition; EHR−, Electronic health records did not contain the documentation of the condition.

**Table 4:** Feature importance from supervised machine learning models comparing survey and electronic health record (EHR) reporting patterns using the concordant positive group (EHR+/Survey+) as the reference for traumatic brain injury (TBI) and colorectal cancer.

	TBI		Colorectal Cancer	
Predictors	EHR+/Survey− ^a^	EHR−/Survey+ ^b^	EHR+/Survey− ^c^	EHR−/Survey+ ^d^
Age	0.22	0.25	0.28	0.26
Education	0.19	0.19	0.25	0.16
Insurance	0.19	0.20	0.18	0.23
Race	0.30	0.20	0.16	0.19
Sex	0.10	0.16	0.12	0.16

^a^, AUC = 0.71; ^b^, AUC = 0.53; ^c^, AUC = 0.59; ^d^, AUC = 0.54.

## Data Availability

The datasets presented in this article are not readily available because they are derived from the All of Us Research Program and are subject to institutional data use agreements, researcher training, and privacy protections that restrict public sharing. Requests to access the datasets should be directed to All of Us research program team (All of Us Research Hub, https://researchallofus.org/).

## Supplementary Materials

The following supporting information can be downloaded at: https://www.mdpi.com/article/10.3390/healthcare14101337/s1, Figure S1. Cohort building process in the *All of Us* Researcher Workbench for traumatic brain injury (TBI) and colorectal cancer.

## Abbreviations

The following abbreviations are used in this manuscript:

AoU	All of US
AUC	area under the receiver operating characteristic curve
CI	Confidence interval
EHR	Electronic health record
NIH	National Institutes of Health
NPV	Negative Predictive Value.
aOR	Adjusted odds ratio
PPV	Positive Predictive Value.
TBI	Traumatic brain injury
VA	Veteran affairs
