# Supplementary material for: Discordance Between Electronic Health Records and Self-Reported Data: Evidence from Traumatic Brain Injury and Colorectal Cancer
Source: Healthcare (Basel). 2026 May 13;14(10):1337. doi: 10.3390/healthcare14101337 (PMC13206625; doi:10.3390/healthcare14101337)

Figure S1. Cohort building process in the *All of Us* Researcher Workbench for traumatic brain injury (TBI) and colorectal cancer. Participants were first identified using survey-based criteria defined by logical "OR". These were then combined with the "Has EHR Data" criterion using a logical AND to include only participants with linked EHR data.

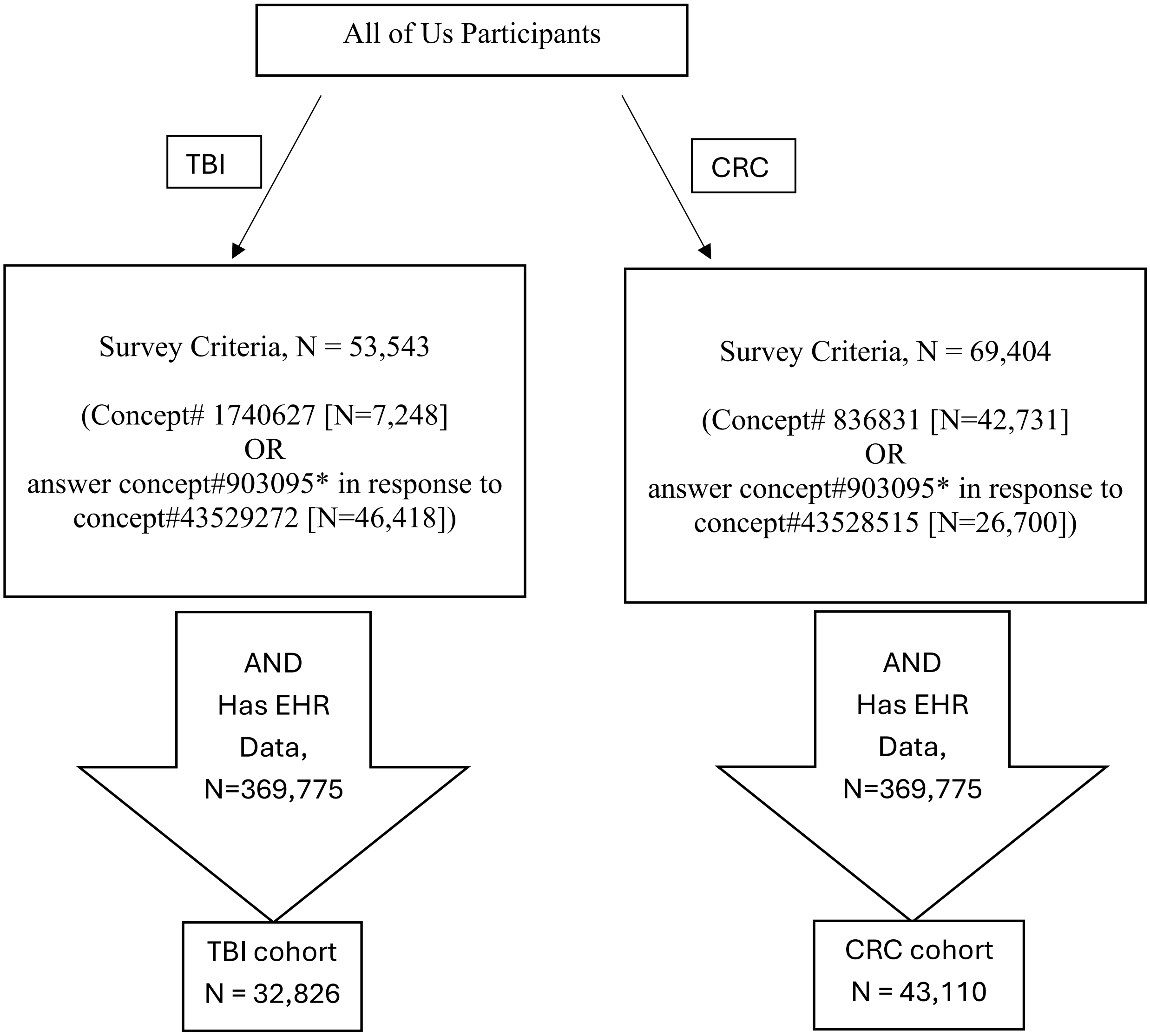

Supplement: Supplementary file 1 [file healthcare-14-01337-s001.zip › healthcare-4294593-supplementary.pdf]
